# The Isotopic Signature of Lead Emanations during the Fire at Notre Dame Cathedral in Paris, France

**DOI:** 10.3390/ijerph18105420

**Published:** 2021-05-19

**Authors:** Philippe Glorennec, Aurélia Azema, Séverine Durand, Sophie Ayrault, Barbara Le Bot

**Affiliations:** 1 Irset (Institut de Recherche en Santé, Environnement et Travail)—UMR_S 1085, Inserm, École des Hautes Etudes en Santé Publique (EHESP), University of Rennes, F-35000 Rennes, France; severine.durand@ehesp.fr (S.D.); barbara.lebot@ehesp.fr (B.L.B.); 2 Laboratoire de Recherche des Monuments Historiques, UMR 3224, F-77420 Champs-sur-Marne, France; aurelia.azema@culture.gouv.fr; 3 Laboratoire des Sciences du Climat et de l’Environnement/CEA-CNRS-UVSQ, UMR 8212, Université Paris Saclay, 91191 Gif-sur-Yvette, France; sophie.ayrault@lsce.ipsl.fr

**Keywords:** isotope, fingerprint, lead poisoning, environmental health, pollution

## Abstract

When Notre Dame de Paris cathedral caught fire on 15 April 2019, lead particles were deposited in its surroundings. Our objective was to determine whether the lead plume had a homogeneous isotopic signature (i.e., a set of homogenous isotopic ratios), and whether, if so, this was different from common sources. In January 2020, dust samples were collected from six areas inside the cathedral, downwind of the fire, as well as from eight roof debris fragments. These samples were mineralized and analyzed using ICP-MS. Their isotopic ratios (207Pb/206Pb and 206Pb/204Pb) were determined and then compared both to each other and to previous published ratios measured in home dusts and blood samples collected in France. The isotopic ratios of dust samples collected inside the cathedral were compatible with each other and with the roof fragments. These isotopic ratios are common and differ neither from those of many other dusts collected in France during the period 2008–2009, nor from those of blood samples collected from children in France during the same period. Moreover, the fire’s isotopic signature is close to the overall signature for Paris. Indeed, it would be difficult to attribute the fire at the cathedral to either lead poisoning or environmental contamination.

## 1. Introduction

On 15 April 2019, Notre Dame cathedral, located in the very center of Paris (France), caught fire. Lead particles were emitted from the cathedral as the main part of the lead roof and spire melted, flowed, and then solidified again inside the building. Its quantity has been estimated at 140 kg [1], and at about 1000 kg by Van Geen [2]. After melting, this lead was aerosolized in the plume; because of the high temperature (above 600 °C), the particles were oxidized—which is why the plume was yellow. Then both the cathedral (inside and outside) and its surroundings were covered with dust that contained a high concentration of lead. The dust layer was thickest in the west part of the building, especially close to the organ pipes [3]. Many environmental samples, and many people, were tested for lead concentration levels in the weeks and months following the event. Since the concentration cannot stand alone as proof of the origin of the lead, the Paris Region Prefect and health agency announced a study to verify whether lead from the cathedral roof is specific or not in its isotopic composition. If it were, this would provide a signature of lead from the cathedral that could be compared to other sources of lead identified in environmental or biological samples [4]. Isotopic fingerprinting is currently used in various domains to identify a source [5], especially for lead poisoning [6], and notably in France [7].

This study’s objectives are firstly to determine whether the isotopic signature of the lead plume is homogeneous, and secondly to determine whether it is singular, i.e., different from common lead sources and human exposures.

## 2. Materials and Methods

### 2.1. Study Design

**Figure 1:**
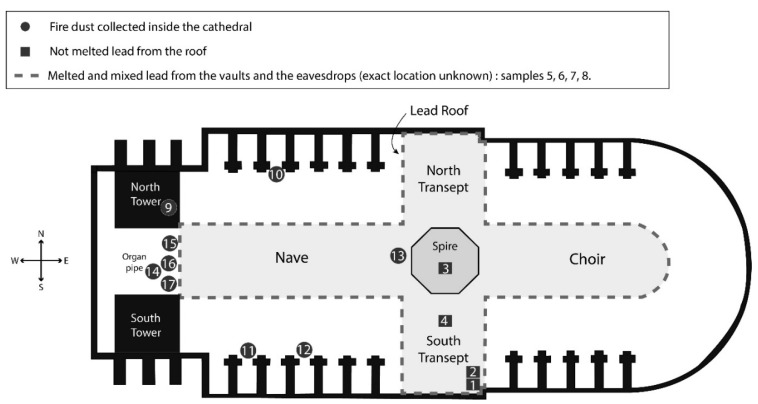
Location and nature of the samples, Notre Dame de Paris cathedral, 2020.

On 31 January 2020, eight fallen fragments of the former roof and spire were sampled (four of melted and mixed lead, and four of lead not melted), as well as nine dust samples from inside the cathedral, downwind of the fire—thus considered fire dust. The locations of these samples are indicated in Figure 1. Although samples 1 and 2 are located close to each other, one is from a roof sheet, probably dated from the XIXth century; the other one is from a valley gutter dated from a restoration in the XXth century.

### 2.2. Sample Collection

Dust samples (*n* = 8) were sampled using a moist, lead-free wipe (“Lead Wipe” by Aramsco) that meets ASTM© standard E1792-03E1792-03. One dust sample (no.14) was sampled using a paintbrush and placed into a tube. Pieces of the roof and spire (*n* = 8), were sampled using tweezers. All samples were stored in polypropylene tubes at room temperature.

### 2.3. Sample Analysis

#### 2.3.1. Materials and Reagents

Solutions of 67% nitric acid and 34% hydrochloric acid (NORMATOM) were supplied by VWR International. Ultra-high quality (UHQ) water was produced in the lab using a Milli-Q gradient water purification system obtained from Millipore, with a resistivity of 18.2 MΩ/cm at 25 °C. The standard solutions of each element at 1 g/L in nitric acid solution were supplied by Techlab. The standard reference material (1640a), manufactured by the National Institute of Standards and Technology (NIST), was used to check the detector linear range. Two certified materials of elements in dust (NIST 2583) and one in soil (CRM SS1, product by ScP Science, Courtaboeuf, France) were used to control the mineralization. The isotopic ratios were corrected using standard reference material NIST 981 (standard high purity lead metal, of 99.9+ percent purity) commercialized into wire form. The precision balance (MSE225P-100-DA), the ultrasonic bath (Elmasonic S120), and the graphite block digestion system (Digiprep) were provided respectively by Sartorius (Dourdan, France), Grosseron (Coueron, france) and ScP Science. A quadrupole ICP-MS (Agilent Technology mass spectrometer, 7900 inductively coupled plasma) equipped with a quadrupole mass filter was used for lead quantification and isotopic ratio evaluation. This system was equipped with an autosampler ASX 500, a micro nebulizer (micro flow), and a nebulizing Scott chamber (all product by Agilent Technology, Paris, France). The ICP-MS settings were as follows: sample rate of 200 µL/min, RF power of 1550 W, a chamber temperature of 2 °C, and a plasma gas flow rate of 15 L/min. The make-up gas flow rate was 0.3 L/min and the nebulizer gas flow rate was 0.8 L/min. 

#### 2.3.2. Sample Pre-Treatment

All tubes containing dust sample wipes were placed in a graphite block and then dried at 50 °C, with a watch glass on the top. After 12 h, the dust was isolated and collected in a second 50 mL polypropylene tube. Each piece of roof or spire was washed, using a sequential four-step method with different solutions (ultrapure water, aqua regia 1/3 HNO_3_ 67% and 2/3 HCl 34%), acidified water at 1% HNO_3_, and ultrapure water. The objective was to remove any deposits from their surfaces. Each piece was placed in a tube containing a liquid, then agitated in the ultrasound bath for a period of 15 min. Nitrogen was then used to dry the samples.

#### 2.3.3. Lead Solubilization 

A mass of around 10 to 200 mg of all samples or standard reference materiel (SRM 981) was weighed and solubilized using 20 mL nitric acid at 67%. After closing the tubes with screw caps, the samples were placed in the ultrasonic bath for 30 min. After 24 h in contact with nitric acid at ambient temperature, samples went to a second ultrasonic bath for 30 min before being diluted to 50 mL with ultrapure water. After sample filtration (Whatmann 110 diameter, ref 1440-110, previously rinsed with acidified water), all samples were diluted in aqua regia solution to obtain a lead concentration of around 10 µg/L for analysis. 

#### 2.3.4. ICP/MS Analysis

A first sample analysis was carried out to determine the lead concentration in each sample. An internal standard mixture (Ir, isotope 193) was added on-line to all samples to verify the absence of ICP-MS signal drift, and for the quantification of all samples. The calibration range for lead was checked with the reference material Nist 1640. At the solubilization and analysis steps, two blanks and three controls (NIST 2583 and CRM SS1) were added. A second sample analysis was used to determine the isotopic ratio (IR). All samples and controls were analyzed at the same concentration (10 µg/L) to improve signal precision on each isotope (relative standard deviation, RSD < 0.3%). The various isotopes (204, 206, 207, and 208) were measured five times on each sample for a sweep at 500. Integration times were 22.5 s for 204 Pb, 13.5 s for 206 Pb and 207 Pb, and 4.5 s for 208 Pb. A correction equation was used to take the 204 Hg mass mercury interferences into account. 

To minimize signal deviation between sequential measurements of the various isotopes, acquisition time was optimized by calibrating dead time with a specific element (Erbium). Two erbium solutions (50 µg/L and 1 mg/L) were prepared to calibrate dead time. First, the sensitivity of the solution at 50 µg/L is checked. Calibration of the dead time was carried out after passing the least concentrated Er solution and then the most concentrated Er solution. The observed value was 35.6 ns.

For the isotopic ratio measurement, the bracketing method [8] was used. The objective was to correct mass bias using a certified reference material for each sample (Common Lead Isotopic Standard, SRM 981) and evaluating the standard deviation after subtraction of the abundances of the analytical blanks. Isotopic ratios (IR) and standard deviations (SD) were calculated using the standard deviations of the certified standards analyzed before and after each sample, following Equations (1) and (2).

Equation (1):

IR = IR sample × (IR Std θ)/((√(IRStd1)) × (√(IRStd2)))(1)

Equation (2):

SD = √((SD sample)^2 + (0.5 × SD Std1)^2 + (0.5 × SD Std2)^2) (2)

With:

- IR—Isotopic Ratio;
- IR sample—uncorrected Isotopic Ratio;
- IR Std θ—Isotopic Ratio of SRM 981 certified;
- IR Std1—Isotopic Ratio of SRM 981 analyzed before the sample;
- IR Std2—Isotopic Ratio of SRM 981 analyzed after the sample;
- SD—Standard deviation;
- SD sample—Uncorrected standard deviation;
- SD Std 1—Standard deviation of SRM 981 analyzed before the sample;
- SD Std 2—Standard deviation of SRM 981 analyzed after the sample.

### 2.4. Isotopic Ratio Interpretation

A figure of merits for the determination of the lead isotopic compositions of the certified material SRM-981 (NIST, Metz, France) and lead isotopic ratio of CRM SS1 (SCP Science) and SRM 2583 (NIST) materials are presented in the Supplemental Materials. The comparison of isotopic signatures was performed on graphs using the isotopic 207 Pb/206 Pb and 206 Pb/204 Pb ratios, as this had previously been demonstrated as the more discriminant in the French context [9]. Uncertainty in measurement is represented on graphs by error bars (±two standard deviations around isotopic ratios). To assess whether the signature of the fire is homogeneous, the isotopic signatures of dust were compared to one another, taking uncertainties into account. The isotopic signatures of dust were also compared to the roof and spire signatures, to assess consistency. The dust signatures were also compared to published data representative of children living in France in 2008–2009 [9]. Children having a blood lead level equal to or above 25 µg/L had lead isotopic measurements both in their blood and in settled dust in their homes.

## 3. Results

**Figure 2:**
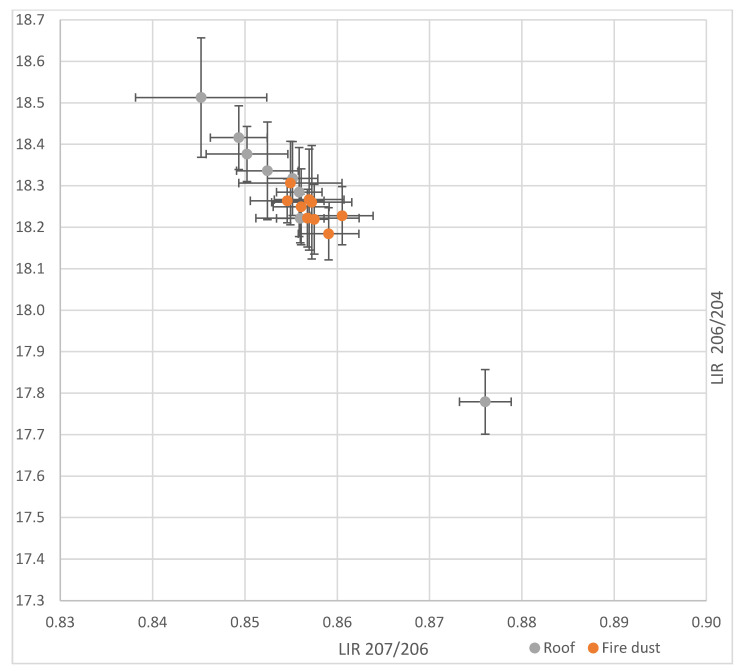
Lead isotopic ratios of Notre Dame de Paris cathedral roof and of dust from its fire.

The lead isotopic ratios of the cathedral roof, and of its dust from the fire, are shown in Figure 2 and displayed in the Supplemental Materials. The roof parts having the lowest Pb207/206 and the highest Pb206/204 (closest to the top left corner) are those of the roof table from the southern transept (No.1). The roof part having the highest Pb207/206 and lowest Pb206/204 (closest to the bottom right corner) corresponds to the roof valley of the cathedral’s southern transept (east face) (No.2). Figure 2 shows that most of the roof and spire plots of isotopic ratios, with their associated measurement uncertainties, overlap, with the exception of the roof valley of the southern transept (East face). The isotopic ratios of the dusts and their uncertainties are all overlapping. They are consistent with those of the roof, with the exception of the roof valley of the southern transept (East face).

**Figure 3:**
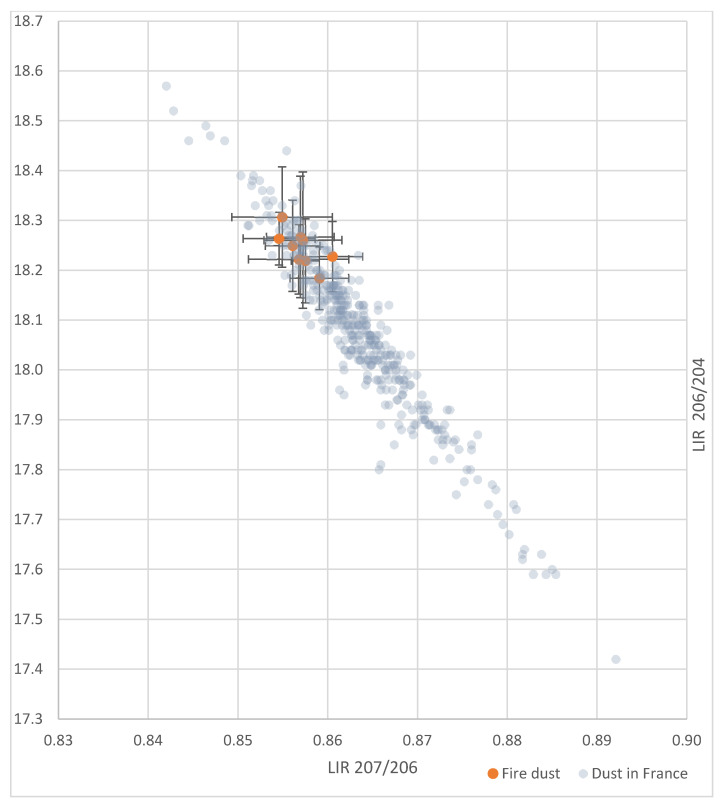
Lead isotopic ratios of Notre Dame de Paris cathedral fire dust and home dust in France.

Figure 3 shows lead isotopic ratios for cathedral fire dust and home dust in France. It indicates that the fire dust IR, with uncertainties, overlaps with many dust lead IRs measured in the homes of young children living in France in 2008–2009, under similar sampling and IR determination conditions [9].

**Figure 4:**
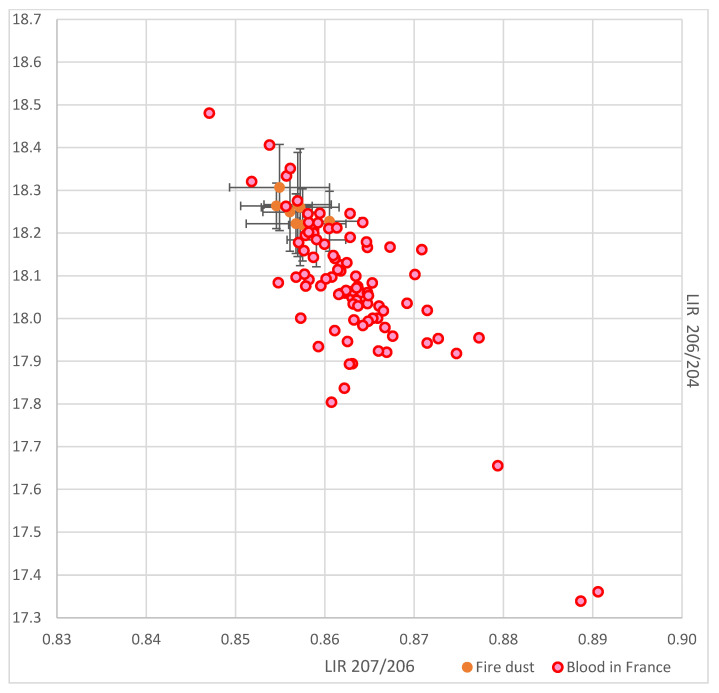
Lead isotopic ratios of Notre Dame de Paris cathedral fire dust and children’s blood in France.

Figure 4 shows the lead isotopic ratios of cathedral dust samples and those of blood from young children living in France in 2008–2009 [9]. It indicates that the dust IRs overlap with several lead IRs that were measured 10 years before the fire, in similar sampling and IR determination conditions.

## 4. Discussion

We assessed the isotopic signature of the dust collected in the cathedral after the fire as being homogeneous, but not source-specific.

Eight dust samples were collected to characterize dust potentially issuing from the fire. These were collected inside the cathedral, downwind, West of the transept. To ensure representativeness of these samples, they were not collected from the ground (because of possible pollution via tracked lead), nor collected from objects possibly containing lead. The homogeneity of the isotopic signature of the dust samples displayed in Figure 2 suggest a single source, namely the fire. This observation is corroborated by the fact that the fire dust signatures lie within the range of variation of those of the majority of roof samples—though the number of roof samples is too small to draw definitive conclusions, especially about roof sheets. It therefore seems apt to consider the existence of an isotopic signature of the fire. The fire dust 206 Pb/207 Pb signature was thus assessed as the arithmetic mean of the 206 Pb/207 Pb in dust and was 1.167 ± 0.005. However, it has to be mentioned that the measurement technique has been chosen not for its precision [10], but for its ability to quickly carry out an isotopic screening on sources and potentially the blood of exposed individuals.

For comparability of analytical techniques and uncertainty of measurements, the results were compared with those of studies carried out in France on dust, soil or sediment, and implemented using the same method (bracketing) and the same type of instrument (quadrupole ICP/MS), with similar performance.

We are not currently aware of any representative assessment of blood lead isotopic signatures in Paris alone. We compared the fire signature with those of dust and blood observed in France in 2008–2009 [9]. We were not able to narrow our analysis to those children issuing from the nationwide study and living in Paris, because very few such children were included in the nationwide study. We did not observe any singularity of the fire dust signature in comparison with French dust and blood.

One practical implication is that a post-fire comparison of the isotopic signatures of blood or dust bearing the signature of the ND de Paris fire would only allow assessment of the cathedral fire’s responsibility for environmental contamination or human exposure if both the pre-fire and post-fire isotopic signatures were (a) known and (b) different. Indeed, as the signature is a common one (shared by much dust and blood in France), it would be difficult to attribute a given case of lead poisoning or a given environmental contamination to the cathedral fire, because many other potential lead sources (paints, soils, dust, water [9]) have signatures similar to that of the fire dust.

The Ayrault et al. study [11] determined typical Paris 207 Pb/206 Pb ratios for sediment cores collected in the basin of the River Seine and dated between 1916 and 2003. Between 1920 and 1960, these ranged from 1.163 ± 0.003 up to 1.166 ± 0.003; between 1960 and 1986/1989, they fell to 1.144 ± 0.003; and after 1990, a rise was observed, to 1.166 ± 0.003 in 2003. The fire dust 206 Pb/207 Pb signature, at 1.167 ± 0.005, cannot be distinguished from the signature of the present-day River Seine sediments. This suggests that the lead which contaminates the Seine River sediments and the lead emitted by the fire have originated from the same mine. The River Seine sediment signature prior to 1960 and post-2000 (i.e., before and after the period of leaded gasoline use) is itself close to the signature of Rio Tinto mine (in Spain), which was heavily exploited during the second half of the 19th century (i.e., during the building of the large water lead pipe network of Paris), defined as 206 Pb/207 Pb = 1.1634 ± 0.0001 [11]. This mine could have provided most of the lead used for the renewal of the roof of the cathedral in the 19th century. This is consistent with the recent study by Smith et al. [12], which observed that the fire did not measurably alter the isotopic composition of Parisian honey, despite an increase in concentrations in honey downwind of the cathedral. These observations are the beginning of a full, in-depth historical study in its own right. Given that since it was first built, the lead roof has been entirely renovated at least twice (18th and 19th centuries), and the fact that many partial renovations have been undertaken before, between and after those major renovations [13], it is in need of contextualization. Usually, the lead from the previous roof was recycled, with the addition of some ‘new lead’ to cast the new sheets. Moreover, since the most recent complete renewal in the 19th century, some localized parts of the roof have also been repaired or altered (eavesdrop, roof valley, roof top ornament, etc.) and correspond to the outlier measurement (sample No.1), which comes from a recently renovated part of the roof. The study of the use of lead in the construction of the cathedral is the subject of a current parallel research program being conducted by the CNRS and the French Ministry of Culture.

## 5. Conclusions

The fire’s isotopic signature was assessed from the dust collected inside the cathedral, since no adequate material was timely sampled outside. It is close to the overall signature for Paris. Indeed, it would be difficult to attribute the fire at the cathedral to either lead poisoning or environmental contamination.

## Data Availability

See supplemental information.

## Supplementary Materials

The following are available online at https://www.mdpi.com/article/10.3390/ijerph18105420/s1: Table S1, figure of merits for the determination of the lead isotopic compositions of the certified material SRM-981; Table S2, roof and dust samples’ lead isotopic composition.
