# Supplementary material for: The Isotopic Signature of Lead Emanations during the Fire at Notre Dame Cathedral in Paris, France
Source: Int J Environ Res Public Health. 2021 May 19;18(10):5420. doi: 10.3390/ijerph18105420 (PMC8159146; doi:10.3390/ijerph18105420)
Supplement: Supplementary file 1 [file ijerph-18-05420-s001.zip › ijerph-1209084-supplementary.pdf]

Article

The Isotopic Signature of Lead Emanations during the Fire at Notre Dame Cathedral in Paris, France

Philippe Glorennec [1,*], Aurélia Azema [2], Séverine Durand [1], Sophie Ayrault [3] and Barbara Le Bot [1]

[1] Irset (Institut de Recherche en Santé, Environnement et Travail)—UMR_S 1085, Inserm, École des hautes études en santé publique (EHESP), University of Rennes, F-35000 Rennes, France; severine.durand@ehesp.fr (S.D.); barbara.lebot@ehesp.fr (B.L.B.)
[2] Laboratoire de Recherche des Monuments Historiques, UMR 3224, F-77420 Champs-sur-Marne, France; aurelia.azema@culture.gouv.fr
[3] Laboratoire des Sciences du Climat et de l'Environnement/CEA-CNRS-UVSQ, UMR 8212, Université Paris Saclay, 91191 Gif-sur-Yvette, France; sophie.ayrault@lsce.ipsl.fr
* Correspondence: philippe.glorennec@ehesp.fr

Supplemental information

Table S1. Figure of merits for the determination of the lead isotopic compositions of the certified material SRM-981 (NIST) and lead isotopic ratio of CRM SS1 (SCP Science) and SRM 2583 (NIST) materials.

	Standard Reference Material 981 (Certified Isotopic Ratio)						Standards Materials (Not Certified Isotopic Ratio)	
**Pb Isotopic Ratios**	**Average after Correction with Bracketing Method (*n* = 20)**	**Average without Correction with Bracketing Method (*n* = 20)**	**SRM981 Certified Values**	**Mean Mass Bias with Correction**	**Mean Mass Bias without Correction**	**Contribution of Analytic Blank on Ratios**	**CRM SS1 (Soil) Average after Correction with Bracketing Method (*n* = 1)**	**SRM 2583 (Dust) Average after Correction with Bracketing Method (*n* = 1)**
**207/206**	0.914	0.898	0.915	0.000	0.017	0.43%	0.823	0.904
Standard deviation (SD)	0.002	0.002					0.002	0.002
RSD (%)	0.21	0.17						
**208/206**	2.168	2.122	2.168	0.000	0.046	0.41%	2.023	2.149
Standard deviation (SD)	0.006	0.004					0.004	0.008
RSD (%)	0.27	0.21						
**206/204**	16.944	17.125	16.937	-0.007	-0.188	0.46%	19.070	17.159
Standard deviation (SD)	0.037	0.033					0.025	0.034

RSD (%)	0.22	0.19						
**208/207**	2.371	2.364	2.370	0.000	0.006	0.43%	2.458	2.377
Standard deviation (SD)	0.006	0.004					0.005	0.009
RSD (%)	0.25	0.18						
**207/204**	15.494	15.373	15.491	-0.003	0.118	0.47%	15.693	15.510
Standard deviation (SD)	0.034	0.028					0.023	0.034
RSD (%)	0.22	0.18						
**208/204**	36.731	36.346	36.721	-0.009	0.375	0.46%	38.570	36.868
Standard deviation (SD)	0.103	0.078					0.075	0.134
RSD (%)	0.28	0.21						

Table S2. Roof and dust samples lead isotopic composition.

Pb Isotopic Ratios	Roof_1	Roof_2	Roof_3	Roof_4	Roof_5	Roof_6	Roof_7	Roof_8	Dust_1	Dust_2	Dust_3	Dust_4	Dust_5	Dust_6	Dust_7	Dust_8	Dust_9
**207/206**	0.8552	0.8760	0.8453	0.8524	0.8502	0.8560	0.8493	0.8559	0.8570	0.8605	0.8568	0.8549	0.8546	0.8572	0.8561	0.8575	0.8591
2 × SD 207/206	0.0027	0.0028	0.0071	0.0034	0.0044	0.0026	0.0031	0.0025	0.0038	0.0034	0.0056	0.0056	0.0040	0.0043	0.0030	0.0016	0.0033
**208/206**	2.1540	2.1242	2.0844	2.0968	2.0952	2.1006	2.0917	2.0946	2.0978	2.2258	2.0998	2.0959	2.1150	2.0982	2.0984	2.1036	2.1034
2 × SD 208/206	0.0155	0.0076	0.0171	0.0090	0.0127	0.0132	0.0111	0.0171	0.0190	0.0116	0.0173	0.0103	0.0930	0.0099	0.0104	0.0149	0.0112
**206/204**	18.3175	17.7791	18.5128	18.3362	18.3764	18.2213	18.4161	18.2847	18.2667	18.2276	18.2220	18.3067	18.2636	18.2603	18.2492	18.2191	18.1842
2 × SD 206/204	0.0889	0.0778	0.1441	0.1177	0.0664	0.0586	0.0769	0.1074	0.1218	0.0701	0.0698	0.1007	0.0532	0.1368	0.0917	0.0841	0.0630
**208/207**	2.5189	2.4248	2.4660	2.4598	2.4643	2.4540	2.4627	2.4473	2.4479	2.5866	2.4508	2.4515	2.4748	2.4476	2.4511	2.4532	2.4485
2 × SD 208/207	0.0175	0.0075	0.0160	0.0110	0.0138	0.0146	0.0133	0.0169	0.0207	0.0162	0.0146	0.0133	0.1035	0.0117	0.0102	0.0151	0.0139
**207/204**	15.6644	15.5751	15.6479	15.6304	15.6241	15.5973	15.6412	15.6494	15.6538	15.6854	15.6121	15.6508	15.6077	15.6534	15.6229	15.6230	15.6214
2 × SD 207/204	0.0563	0.0765	0.0588	0.0680	0.0919	0.0513	0.0666	0.1160	0.0766	0.0595	0.0622	0.0839	0.0751	0.0919	0.0831	0.0631	0.0443
**208/204**	39.4568	37.7665	38.5873	38.4479	38.5025	38.2758	38.5205	38.2992	38.3188	40.5712	38.2628	38.3684	38.6266	38.3133	38.2937	38.3257	38.2495
2 × SD 208/204	0.2563	0.1870	0.1699	0.2499	0.2019	0.1805	0.1907	0.4833	0.1932	0.2459	0.2832	0.2168	1.6670	0.3585	0.2019	0.2551	0.2081
